# Supplementary material for: An AI-assisted morphoproteomic approach is a supportive tool in esophagitis-related precision medicine
Source: EMBO Mol Med. 2025 Feb 3;17(3):441–68. doi: 10.1038/s44321-025-00194-7 (PMC11903792; doi:10.1038/s44321-025-00194-7)
Supplement: Supplementary file 10 — Expanded View Figures [file 44321_2025_194_MOESM10_ESM.pdf]

Expanded View Figures

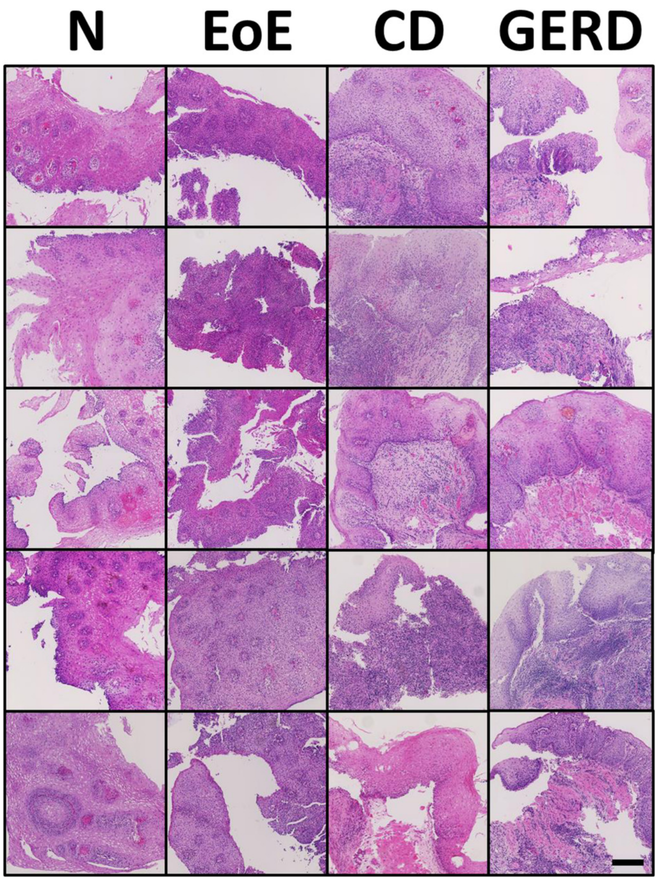

Figure EV1. Histology of reference samples.

Representative micrographs of H&E stains of the reference samples for Normal (*N*), Eosinophilic esophagitis (EoE), Crohn's disease (CD), and gastro-esophageal reflux disease (GERD). Scale bar: 200 µm.

 © The Author(s)

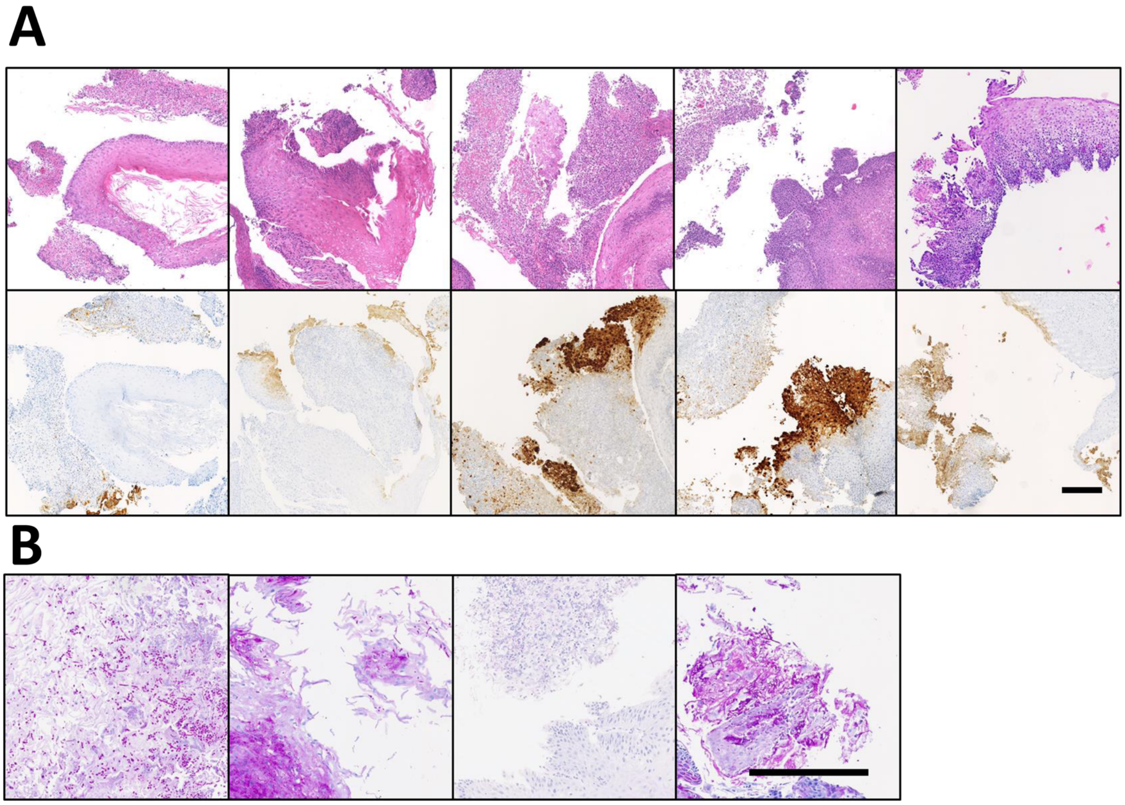

Figure EV2. Histology of infectious samples.

(**A**) H&E stains showing strong inflammation and heterogenous cytopathic changes (top row). IHC stains using an antibody against HSV1/2 highlight HSV-positive cells in brown (bottom row). (**B**) PAS reaction showing pseudo hyphae and spores of Candida spp. The epithelium displaying unspecific changes. Scale bars: 200 µm.

© The Author(s)

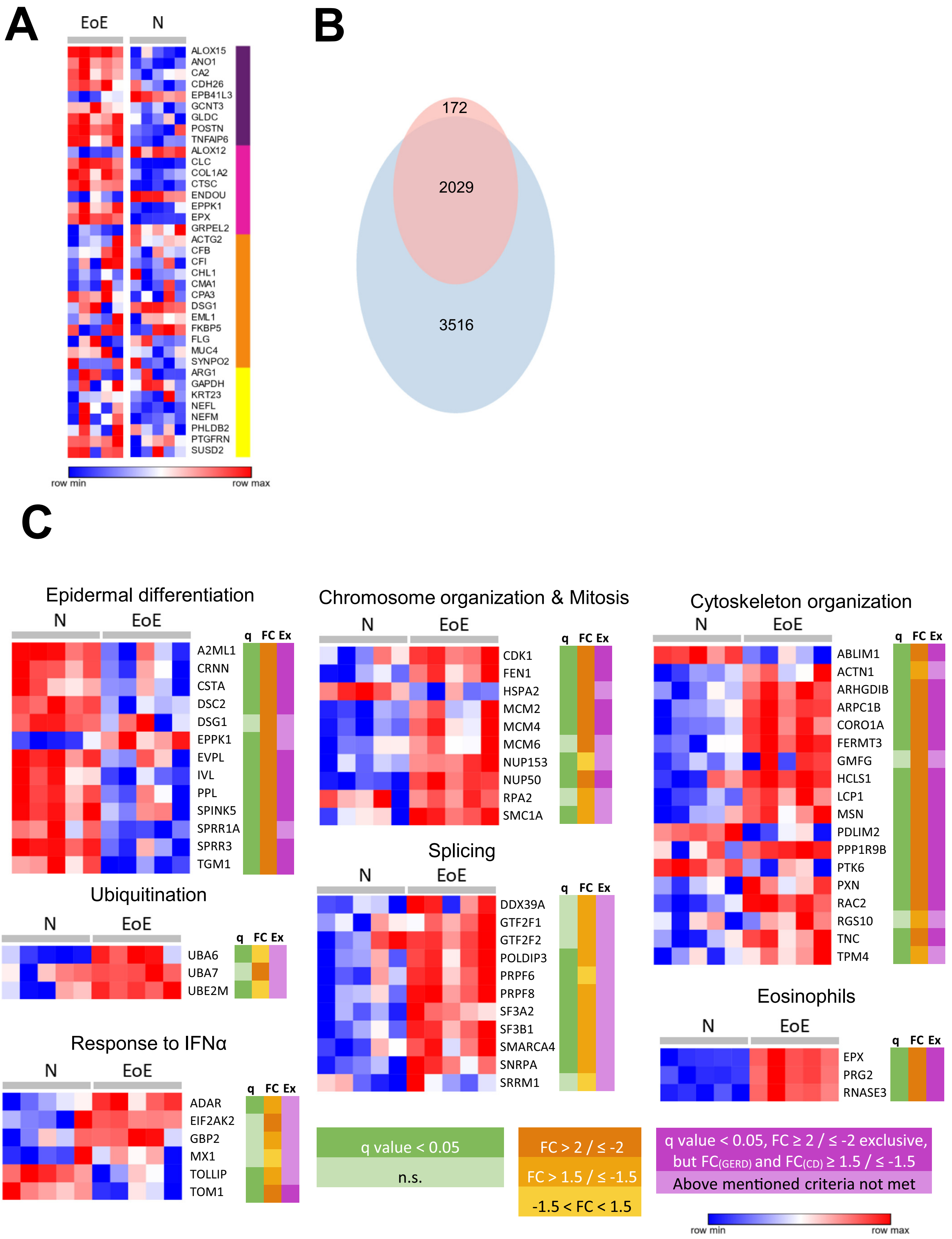
A
EoE
N
ALOX15
ANO1
CA2
CDH26
EPB41L3
GCNT3
GLDC
POSTN
TNFAIP6
ALOX12
CLC
COL1A2
CTSC
ENDOU
EPPK1
EPX
GRPEL2
ACTG2
CFB
CFI
CHL1
CMA1
CPA3
DSG1
EML1
FKBP5
FLG
MUC4
SYNPO2
ARG1
GAPDH
KRT23
NEFL
NEFM
PHLDB2
PTGFRN
SUSD2
row min
row max
B
172
2029
3516
C
Epidermal differentiation
N
EoE
q FC Ex
A2ML1
CRNN
CSTA
DSC2
DSG1
EPPK1
EVPL
IVL
PPL
SPINK5
SPRR1A
SPRR3
TGM1
Ubiquitination
N
EoE
q FC Ex
UBA6
UBA7
UBE2M
Response to IFNα
N
EoE
q FC Ex
ADAR
EIF2AK2
GBP2
MX1
TOLLIP
TOM1
Chromosome organization & Mitosis
N
EoE
q FC Ex
CDK1
FEN1
HSPA2
MCM2
MCM4
MCM6
NUP153
NUP50
RPA2
SMC1A
Splicing
N
EoE
q FC Ex
DDX39A
GTF2F1
GTF2F2
POLDIP3
PRPF6
PRPF8
SF3A2
SF3B1
SMARCA4
SNRPA
SRRM1
Cytoskeleton organization
N
EoE
q FC Ex
ABLIM1
ACTN1
ARHGDIB
ARPC1B
CORO1A
FERMT3
GMFG
HCLS1
LCP1
MSN
PDLIM2
PPP1R9B
PTK6
PXN
RAC2
RGS10
TNC
TPM4
Eosinophils
N
EoE
q FC Ex
EPX
PRG2
RNASE3
q value < 0.05
n.s.
FC > 2 / ≤ -2
FC > 1.5 / ≤ -1.5
-1.5 < FC < 1.5
q value < 0.05, FC ≥ 2 / ≤ -2 exclusive,
but FC(GERD) and FC(CD) ≥ 1.5 / ≤ -1.5
Above mentioned criteria not met
row min
row max

 © The Author(s)

Figure EV3. Subset of proteins quantified in EoE versus N corresponding to previously published markers for EoE.

(**A**) Heatmap showing relative abundances of the indicated proteins in normal (*N*) and eosinophilic esophagitis (EoE) samples. The proteins correspond to markers (mRNAs) which were previously published (Wen et al, 2013) as part of an EoE diagnostic panel (EDP). Purple: exclusive for EoE v N; pink: significant, but not exclusive for EoE v N; orange: significant in CD v N or GERD v N, but not in EoE v N; yellow: not significantly altered, (**B**) Venn diagram of all quantified proteins in our dataset compared to a recent publication on tissue proteomics showing the differences between N and EoE samples (Rochman et al, 2023), (**C**) Heatmaps of the proteins mentioned in the enrichment analysis in Rochman et al, (2023) comparing N and EoE samples. Color coding showing different criteria for significance (q value, FC and exclusiveness in our dataset compared to GERD and CD samples) as stated on the bottom.

© The Author(s)

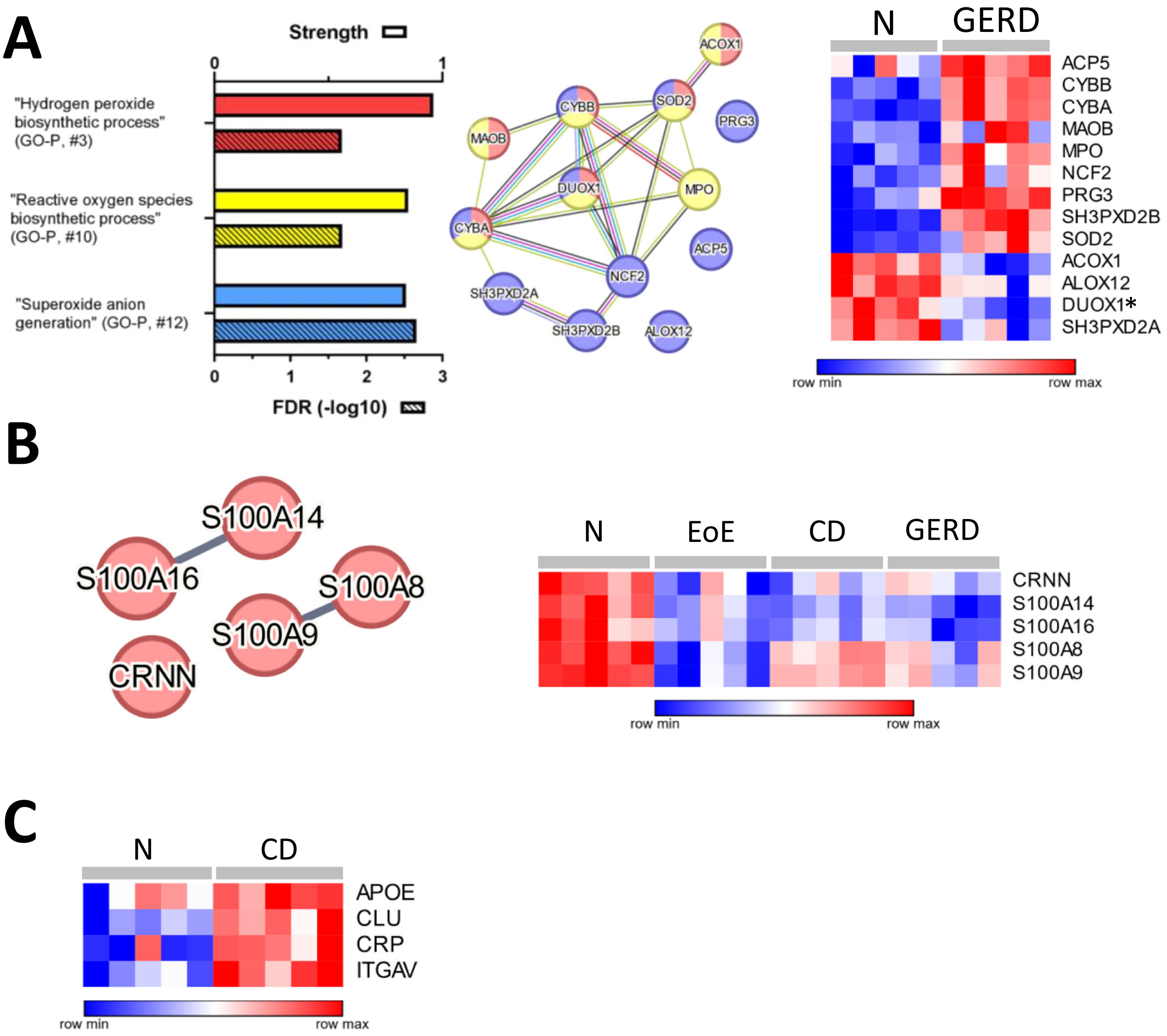

Figure EV4. Other relevant proteins of different comparisons.

(**A**) STRING networks of reactive oxygen species (ROS)-associated proteins and corresponding heatmaps (relative abundances of indicated proteins in each N and GERD sample) as well as related enrichment terms highlighted (for complete list see Dataset EV3B) by bar diagrams with the respective strength and false discovery rate (FDR, -log10, hatched bar) as well as the rank in the respective database. (**B**) STRING networks of S100A- and associated proteins and corresponding heatmaps (relative abundances of indicated proteins in each N, EoE, CD, and GERD sample). All of those proteins are significantly altered between N and each of the inflammatory groups. Except for S100A8 and S100A9 (each in N vs CD) all other proteins exceed log2FC 1/−1 (Dataset EV1C). (**C**) Heatmap (relative abundances of indicated proteins in each N and CD sample) of proteins previously described as predictive markers for anti-TNF-α therapy (Gazouli et al, 2013; Kalla et al, 2021; Kumar et al, 2024). The lowest value in each row in the heatmaps is displayed in dark blue, the highest value in dark red. The scale traverses white. The heatmaps were created using Morpheus.

 © The Author(s)

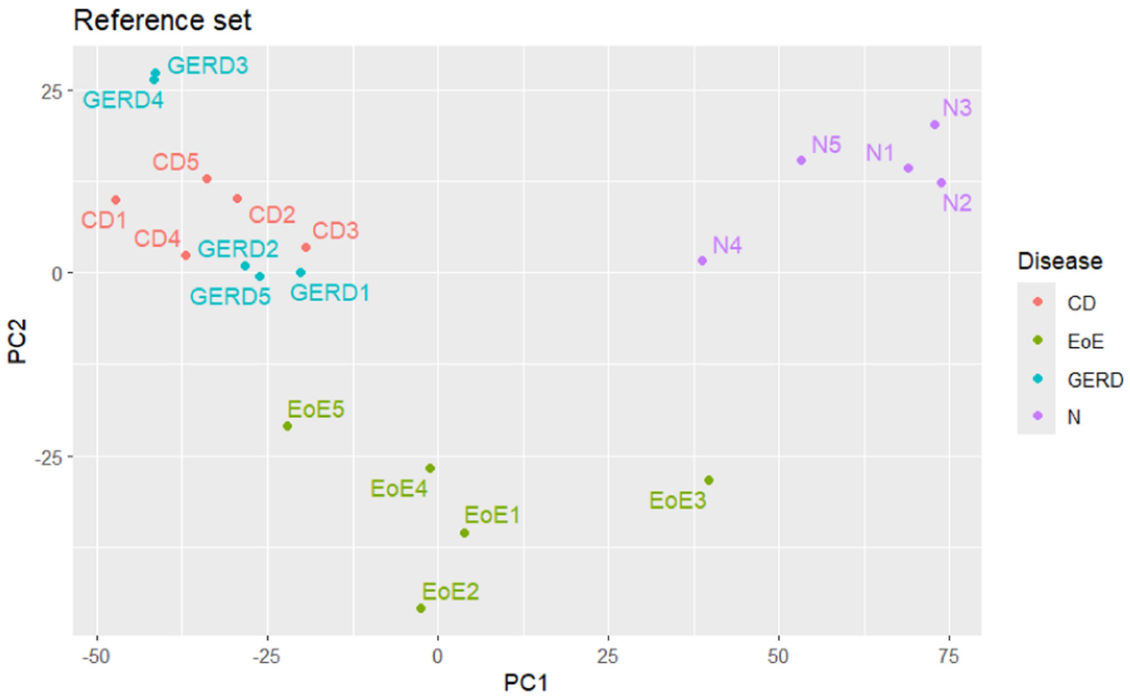

Figure EV5. PCA plot of reference dataset.

Principal component analysis (PCA) performed on the reference dataset illustrates separation of N, EoE, and the mixed cluster of GERD and CD samples.

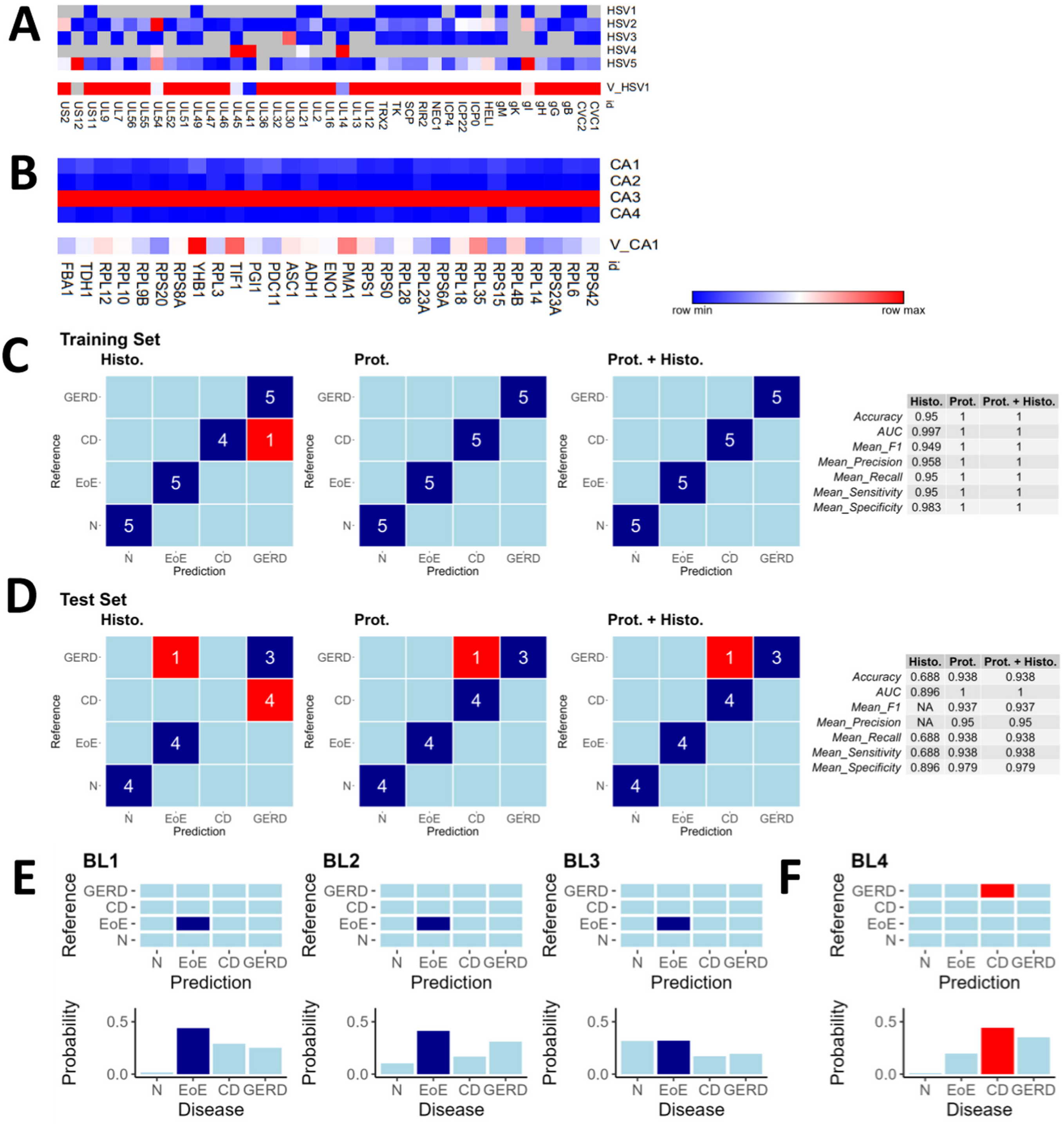

Figure EV6. Validation of proteomic approach in infectious conditions, illustration of the random forest (RF) parameters in training and test samples, as well as results of borderline cases.

(**A**) HSV-specific proteins are detected in the HSV esophagitis test sample (V_HSV1). (**B**) Candida-specific proteins are detected in the Candida esophagitis test sample (V_CA1). The lowest value in each column of the heatmaps is displayed in dark blue, the highest value in dark red. The scale traverses white. Heatmaps were created using Morpheus. (**C**) Results of the RF approach in the training dataset for histological features (Histo.) alone, proteome (Prot.) alone and combined (Prot. + Histo.). (**D**) Results of the RF with the aforementioned conditions in the test cohort. (**E**) Results of testing the approach in different borderline conditions. (**F**) Borderline case of a patient with histology and presentation favor GERD. Squares in the confusion matrices and bars colored in blue indicate the correct classification for each case by the RF, red markings indicating an incorrect classification.

© The Author(s)
